# A model to facilitate implementation of the International Classification of Functioning, Disability and Health into prosthetics and orthotics

**DOI:** 10.1177/0309364617729925

**Published:** 2017-09-14

**Authors:** Gustav Jarl, Nerrolyn Ramstrand

**Affiliations:** 1 Department of Prosthetics and Orthotics, Faculty of Medicine and Health, Örebro University, Örebro, Sweden; 2 University Health Care Research Center, Faculty of Medicine and Health, Örebro University, Örebro, Sweden; 3 CHILD research group, Department of Rehabilitation, School of Health and Welfare, Jönköping University, Jönköping, Sweden

**Keywords:** Orthotics, prosthetics, rehabilitation, International Classification of Functioning, Disability and Health, treatment outcomes

## Abstract

**Background:**

The International Classification of Functioning, Disability and Health is a classification of human functioning and disability and is based on a biopsychosocial model of health. As such, International Classification of Functioning, Disability and Health seems suitable as a basis for constructing models defining the clinical P&O process. The aim was to use International Classification of Functioning, Disability and Health to facilitate development of such a model.

**Proposed model:**

A model, the Prosthetic and Orthotic Process (POP) model, is proposed. The Prosthetic and Orthotic Process model is based on the concepts of the International Classification of Functioning, Disability and Health and comprises four steps in a cycle: (1) Assessment, including the medical history and physical examination of the patient. (2) Goals, specified on four levels including those related to participation, activity, body functions and structures and technical requirements of the device. (3) Intervention, in which the appropriate course of action is determined based on the specified goal and evidence-based practice. (4) Evaluation of outcomes, where the outcomes are assessed and compared to the corresponding goals. After the evaluation of goal fulfilment, the first cycle in the process is complete, and a broad evaluation is now made including overriding questions about the patient’s satisfaction with the outcomes and the process. This evaluation will determine if the process should be ended or if another cycle in the process should be initiated.

**Conclusion:**

The Prosthetic and Orthotic Process model can provide a common understanding of the P&O process. Concepts of International Classification of Functioning, Disability and Health have been incorporated into the model to facilitate communication with other rehabilitation professionals and encourage a holistic and patient-centred approach in clinical practice.

**Clinical relevance:**

The Prosthetic and Orthotic Process model can support the implementation of International Classification of Functioning, Disability and Health in P&O practice, thereby providing a common understanding of the P&O process and a common language to facilitate communication with other rehabilitation professionals.

## Background

The International Classification of Functioning, Disability and Health (ICF) is a classification of human functioning and disability developed by the World Health Organization and provides a common language for describing health and health-related states.^1^ The ICF framework and terminology has become the standard in different areas of healthcare and rehabilitation and is receiving increasing recognition in the field of prosthetics and orthotics (P&O).^2,3^ The increasing number of ICF-related publications in P&O subject areas is an indication of rising acceptance by the field: specific studies have linked the content of clinical outcome measures to ICF,^4^ developed P&O specific core sets^2,5^ and recommended instruments for addressing elements of the ICF.^6–10^ Studies have also used ICF as a framework for reviews,^11,12^ clinical trials^13^ and reported experiences of implementing ICF in clinical practice.^14,15^ Given growing acceptance in many medical and allied health professions and the holistic nature of the concepts contained within the ICF, the authors consider it to provide a useful framework for developing a P&O process model to guide the clinical decision-making process.

Before implementing the terminology and classification of ICF, a basic understanding of the ICF concepts is required. It is also necessary to reflect upon how concepts of the ICF relate to P&O processes. To the authors’ knowledge, only a few studies have utilised the ICF framework as a means conceptualising P&O clinical practice: these studies have focused upon outcome measures for upper limb prostheses,^9^ proposing a model for evaluating ankle foot orthoses^16^ and facilitating the prescription and supply of P&O devices in general.^17^ While the approaches described in the literature to date are useful for their purpose, there is a clear need for a model to define the clinical P&O process using a broad holistic perspective. The aim of this article was to use ICF terminology to facilitate development of such a model.

## ICF

### Conceptual framework

The ICF framework classifies functioning and disability according to a biopsychosocial model of health. This model operates on the premise that disability affects not only body structures and functions but also psychological and social factors and acknowledges that there is interaction between concepts within the framework.^18^ As such, prosthetists/orthotists adopting this approach are encouraged to broaden the scope with which they view health and illness and to consider the multidimensional and interactive nature of all concepts within the ICF. For example, it is no longer sufficient that clinicians measure the success of treatment from a pure biomechanical perspective but equally important to demonstrate whether an intervention gives improvements in other aspects related to the way in which a person lives.^19^

The two parts of the ICF support this broader view. Part 1 addresses functioning and disability, including the concepts of body functions, body structures, activities and participation. Part 2 addresses contextual factors, including the concepts of environmental factors and personal factors.^1^ Table 1 presents these concepts with examples of how they could be considered within P&O clinical practice.

**Table 1.** Parts and concepts of the International Classification of Functioning, Disability and Health (ICF).

Parts	Concepts	Definitions	Coding letter	Examples of relevance in P&O
Part 1: functioning and disability	Body functions	Physiological functions of body systems (including psychological functions)	b	Pain, functions of bones, joints, muscles, movement and gait
	Body structures	Anatomical parts of the body	s	Structures related to movement (bones, joints, muscles, etc.) and skin
	Activity	Executions of a task or an action by an individual	a	Manipulating objects and walking
	Participation	Involvement in a life situation	p	Self-care, household tasks, maintaining P&O devices, interpersonal relationships, education and work
Part 2: contextual factors	Environmental factors	The physical, social and attitudinal environment in which people live and conduct their lives	e	P&O devices, mobility assistive devices, support and attitudes of family and friends
	Personal factors	The particular background of an individual’s life and living	(not coded)	Gender, age, character, experience, interests, profession and lifestyle

P&O: prosthetics and orthotics.

**Figure 1.**
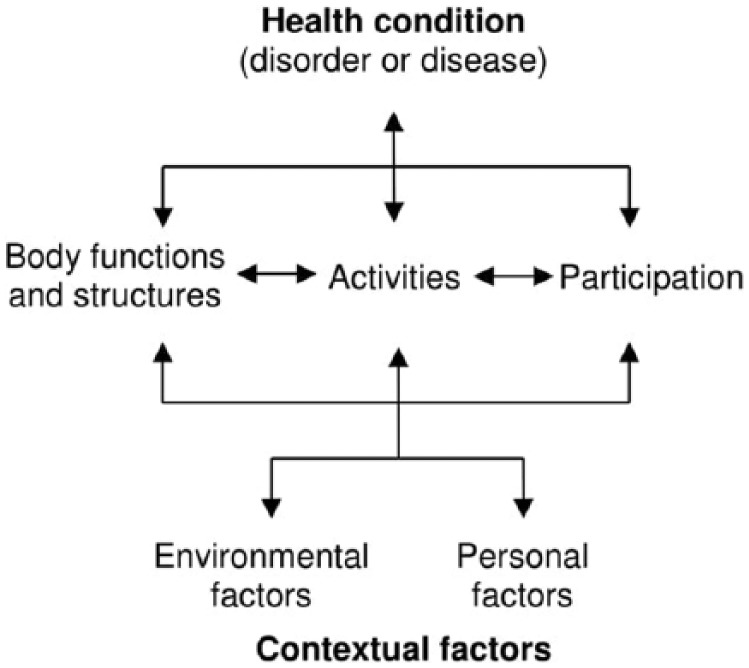
Interactions between concepts of the International Classification of Functioning, Disability and Health (ICF). Reproduced with permission by the World Health Organization.

Different concepts within the ICF are interrelated and individuals’ functioning and disability are conceived as dynamic interactions between their health conditions and contextual factors (Figure 1).

### Coding system

**Figure 2.**
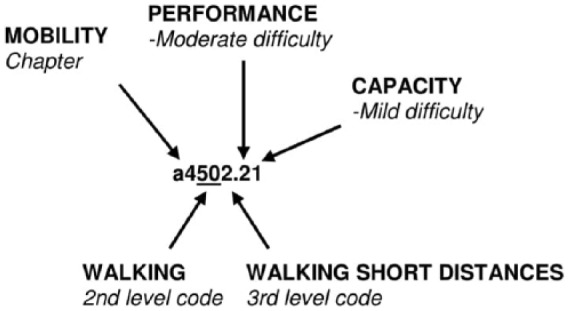
Example on the activity walking including second and third level codes and qualifiers applicable for the ICF category walking short distances. Modified from Pless and Granlund.^20^

The ICF uses a hierarchically organised code system where a letter denotes the ICF concept followed by 1–5 digits which denote the ICF chapter and category levels (Figure 2). The code can be complemented by one or more qualifiers, quantified using a five-level scale (0 = no, 1 = mild, 2 = moderate, 3 = severe/substantial, 4 = complete) and expressing the magnitude of the impairment, severity of the problem and so on. For activities and participation, a first qualifier denotes the level of performance, what the person does in the current environment, including personal support and assistive devices, while a second qualifier denotes the level of capacity, the ability to execute a task in a ‘standard’ environment without support or devices (Figure 2).^1^ It can be appropriate to mention that a third qualifier for participation has been proposed. As participation is defined as ‘involvement in a life situation’, which incorporates ‘being included or engaged in a life area’ (p. 13), there has been a substantial debate that the current performance qualifier does not address the degree of involvement in terms of engagement. It rather describes individuals’ attendance in activities, that is, the frequency with which they participate.^21^

Environmental factors considerably affect participation. They can either be facilitators or barriers, which are distinguished by the sign between the code and qualifier. For example, e460+2 denotes that societal attitudes are a moderate facilitator and e460.2 that they are a moderate barrier. Prostheses and orthoses are classified as environmental factors and are coded as e1151, ‘assistive products and technology for personal use in daily living’.^1^ The ICF describes environmental factors as external to the person and while a prosthesis or orthosis is certainly external, it could be argued that it is much more personal than other aspects of the environment such as ramps or elevators. There has also been some critique regarding the broad categorisation of environmental factors, and additional coding systems have been recommended to be used as a compliment to ICF in order to provide a more detailed classification of P&O devices.^17^

## Functioning in the P&O field

In the proposed Prosthetic and Orthotic Process (POP) model, aspects of the ICF are conceived as different levels of functioning, which should be rated separately and then merged to form the holistic view of a person’s health status. Body functions and structures reflect functioning at the body level. Activities and participation reflect functioning at the individual level, in nine life areas, along a continuum from; limited ability of executing a task independent on the context, to the lived experience of people in their actual context, that is, societal involvement.^1,21,22^ There are different options to separate the concepts activity and participation (see Appendix 3 of ICF^1^). For the purpose of the POP model, we recommend coding chapters 1–4 (learning and applying knowledge, general tasks and demands, communication and mobility) as activities and chapters 5–9 (self-care, domestic life, interpersonal interactions and relationships, major life areas, and community, social and civic life) as participation.

**Figure 3.**
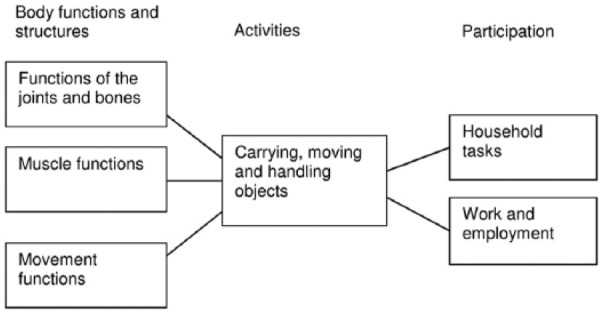
Example of how goals at different levels can contribute to the fulfilment of goals at other levels.

Participation is conceived as the main goal of P&O interventions and is realised by achieving activities-related goals. In a similar fashion, goals related to activities are realised by achieving goals related to body functions and structures. It is important to recognise that different goals on one level can contribute to the fulfilment of the same goal on another level, and one goal on one level can contribute to the fulfilment of different goals on another level (Figure 3).^23,24^ Still, each level needs to be recognised and assessed separately and cannot just be inferred from the other levels.

## The POP model

**Figure 4.**
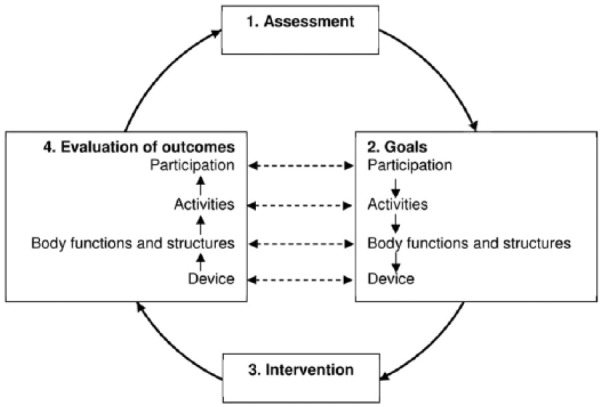
The Prosthetic and Orthotic Process (POP) model.

The POP model was constructed by utilising the ICF and the view of functioning described above. By incorporating concepts from the ICF into the POP model and encouraging use of ICF codes and qualifiers clinicians can ensure that a holistic- and patient-centred approach is used throughout their clinical decision-making process. The POP model comprises four steps in a cycle: assessment, goals, intervention and evaluation of outcomes (Figure 4), which are described below:

1. *Assessment*. The assessment includes medical history and physical examination of the patient. This is based on information about the health condition (coded in ICD-10^25^), body functions, body structures, activities, participation, environmental factors (coded in ICF) and personal factors (not coded in ICF). An ICF-based tool, such as the rehabilitation problem-solving form,^26^ a core set^2,5^ or a check list,^15^ can be used to systematise the information according to the ICF framework. To measure the person’s status in single ICF categories, two approaches can be used. First, the ICF qualifiers described above can be used as a rating scale ranging from 0 to 4 in which all relevant information (from the medical history, physical examination, clinical tests, questionnaires, etc.) is integrated to guide the choice of qualifier. Second, results from specific instruments (clinical tests, questionnaires, etc.) which have previously been linked to ICF can be transformed into a qualifier.^27,28^ The assessment results are thereby translated into the language of ICF, facilitating external comparability of the results.
2. *Goals*. Goals are the objectives of the intervention.^29^ The foremost goals are the participation goals. Once participation goals have been determined, the activity goals, necessary to achieve the set participation goals are specified followed by goals at the body functions and structures level, which is the level that P&O devices operate on. This means that the person’s wishes and values are broken down into their components, until the level where the P&O devices can affect the situation is reached. Given that the technical specifications of a prosthetic or orthotic device are paramount to achievement of specific goals, the POP model specifically incorporates a fourth level for describing technical goals associated with the device. With the exception of technical specifications, the goals at each level can take one of two principal forms, to improve the current state or to prevent or slow a deterioration of the current state (e.g. in case of progressive disease).^30^ These goals are further specified into a specific state that can be quantified using ICF qualifiers. Alternatively, the goal can be quantified in terms of a specific instrument score. Preferably, an instrument that has been linked to ICF should be used so that the score can be transformed into an ICF qualifier. Goals related to technical specifications should describe the desired functional requirements for specific components as well as unique design features (e.g. cosmetic appearance).
3. *Intervention*. An intervention should address the goals and the information gathered in the assessment phase, including personal factors such as the person’s preferences and expectations, as well as environmental factors like the person’s physical environment and social support. The intervention can be a prosthetic or orthotic device, or, in case of an already existing device, adjustment of, or training in, the use of it. Principles of evidence-based practice should be applied when determining the most appropriate intervention.^31,32^ This requires that clinicians weigh up research evidence, clinical experience and patient values when arriving at a clinical decision. When reviewing research evidence, it is important to consider the goals of the intervention, to evaluate how specific research outcomes relate to these goals and to determine if research findings are relevant for the individual patient.
4. *Evaluation of outcomes*. Outcomes are the results of an intervention.^33^ If the person complies with the intervention and the intervention is based on a correct analysis of the situation, there will be outcomes of the intervention at the different levels of functioning. The time frame for the goals and outcomes must be kept in mind. Since P&O devices have a direct effect on the level of body functions and structures and only an indirect effect on the levels of activities and participation, outcomes will often show up in a briefer time period for the former level compared to the latter. To evaluate if the intervention has given the intended effects, that is, if the goals have been achieved, the outcomes are assessed and compared to the corresponding goals at each level. The outcomes can also be compared to the results from the assessment phase to investigate if progress is made towards the goals. No single evaluation method can cover the outcomes at all levels. Rather, different methods are needed and will complement each other to get the full picture.^9,34^ The evaluation methods range from more qualitative methods such as a discussion related to perceived levels of involvement in an activity, to more quantitative methods as kinetic and kinematic gait analysis and other technical measurements. More qualitative methods are appropriate for concepts at the society, and person levels and more quantitative methods are appropriate for concepts at the body level (Table 2). Naturally, the choice of evaluation method depends not only on the concept but also on the specific category at issue. For instance, assessment of phantom limb sensation and gait pattern require different evaluation methods although both belong to body functions. It is however imperative to use the same method for the same category in the assessment and evaluation phases.

**Table 2.** Suggestions for assessment methods (marked with ‘X’) to use for different levels in the Prosthetic and Orthotic Process (POP) model.

	Interviews	Questionnaires	Observations in natural environment	Observations in clinic/function tests	Technical measurements	
Participation	X	X	X			Society/person level
Activities	X	X	X	X	X	
Body functions	X	X	X	X	X	Body level
Body structures				X	X	
Device				X	X	Technical level
	Qualitative methods	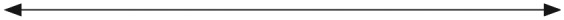			Quantitative methods	

After the evaluation, the first cycle in the process is complete, which brings us back to the assessment phase. A broad assessment is now made including overriding questions about the current situation and the process. Have the goals been reached to a satisfactory degree? Is the person satisfied with the outcomes and the process? Why or why not? Such questions and answers should guide the clinician and the patient in their conjoint decision if the process should be ended or if another cycle in the process should be initiated.

## Patient case

**Figure 5.**
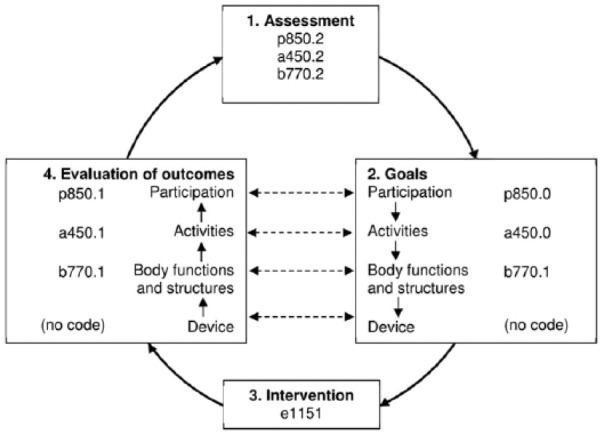
Illustration of a case in the Prosthetic and Orthotic Process (POP) model with codes from the International Classification of Functioning, Disability and Health (ICF). For clarity, not all codes from the text are repeated in the figure.

A fictive case is used to illustrate how the POP model and ICF can be used in clinical practice (Figure 5). Note that the case and process are simplified, and only one qualifier is used in the coding:

1. *Assessment*. A 40-year old male farmer has suffered a traumatic injury of the peroneal nerve resulting in ankle instability, drop foot and a steppage gait deviation, due to loss of muscular control. A core set for lower limb orthoses is used to guide the data collection.^5^ Findings from the patient history, physical examination and visual gait analysis are coded according to ICF. His body functions are affected: ankle stability is severely impaired (body functions, b715.3); he has a complete drop foot (b730.4) and a moderately limping gait (b770.2). This gives him moderate performance problems in his walking (activities, a450.2) and in his work (participation, p850.2). His work as a farmer requires him to walk on uneven ground, serving as a moderate barrier (environmental factors, e210.2).
2. *Goals*. The patient wishes to work as he did before the injury. Thus, the main goal is at the participation level. This can be defined as eliminating his performance problems at work (participation, p850.0). This is achieved by eliminating his performance problems in walking (activities, a450.0), in turn achieved by reducing the limping to a mild level (body functions, b770.1). Durability of the device is considered as a major technical goal and will influence choice of materials in the manufacturing process.
3. *Intervention*. The orthotist reviews current evidence related to orthotic management of drop foot. A systematic review from 2015 was identified and included a review of eight studies involving individuals with dorsiflexion paresis, mainly due to peroneal nerve palsy.^12^ The review indicates that individuals with dorsiflexion paresis benefit more from circular and elastic ankle foot orthoses on outcomes related to energy efficiency but that dorsal (posterior shell), circular and elastic ankle foot orthoses all increased dorsiflexion during swing. Given the patient’s ankle instability and his requirement to walk on uneven ground, the clinician determines, from experience, that a circular or elastic ankle foot orthosis would not address the patient’s performance problems at work and prescribes a carbon fibre ankle foot orthosis (environmental factors, e1151) that can cope with his heavy work as a farmer.
4. *Evaluation of outcomes*. The orthotist gives the person 2 months to adjust to the orthosis so that he can use it full-time at work, thereby making an outcome at all levels possible. The orthosis turns out to be a moderate facilitator (e1151+2). The outcomes are assessed and compared to the goals and the initial assessment. Inspection of the orthosis reveals no sign of material failure indication that the device is sufficiently durable. The goal at the body functions and structures level is achieved, that is, his limping problem has been reduced to a mild level when using the orthosis (b770.1). However, the goals at the activities and participation levels are not reached. He now has a mildly impaired performance in his walking (a450.1) and at work (p850.1), while the goals were to eliminate these performance problems (a450.0 and p850.0). Still, this is an improvement compared to the initial assessment when the performance problems were moderate (a450.2 and p850.2).

### Assessment

In the overall assessment, the person turns out to be satisfied with the outcomes although they were lower than the goals set. The orthotist and the patient agree that the goals were a bit unrealistic facing the demands of his work. Thus, the process is ended.

## Discussion

The ICF has received increasing recognition in rehabilitation practice and scientific publications^35,36^ during the past years and may well fulfil its promise as a universal language for functioning and disability. Still, the implementation in the P&O field is in the early stages, and the potential benefits and issues of implementing ICF in P&O practice are not well known. Studies from other areas of habilitation and rehabilitation suggest that the implementation of the ICF can take considerable time and effort but also can provide substantial gains, such as improved communication, increased awareness of participation goals and contextual factors, and a patient-centred approach.^36,37^ It seems reasonable that these benefits also would come with the implementation of ICF in P&O practice and, thus, the implementation may be well worth the effort.

Different tools can be used to facilitate the implementation of ICF, where each tool fulfils a different need in the implementation process. The POP model could be useful to integrate the ICF conceptual framework and concepts in clinical thinking, which could serve as a foundation to build on for subsequent work with implementing the coding system using core sets,^2,5^ checklists^14,15^ and recommendations about instrument choices.^7–10^ In addition, the POP model could contribute to a common understanding of the P&O process. Together with ICF, this could enhance a stronger theoretical basis for the profession and facilitate the communication between P&O practitioners and other rehabilitation professionals. In clinical practice, the POP model and ICF encourage a holistic and patient-centred approach. In research and development, the POP model and ICF highlight the presence of different levels of goals and outcomes, which all are important to consider.

A model is by definition a simplified representation of reality and, as such, does not include all aspects of the real-life situation. Many patients receive several parallel interventions distributed over different functioning levels and over time, such as surgery to alter body functions and structures and home adaptations to improve the physical environment of the person. Such aspects are not incorporated in the model but should not be forgotten when using it. In addition, the POP model simplifies the process as a unidirectional effect from body functions and structures to activities and participation. In reality, the influence is bi-directional; improved body functions and structures can facilitate activities and participation, but increased activities and participation can also lead to improvement of body functions and structures.^38^ Furthermore, no classification system is perfect and neither is ICF. For instance, there are issues related to how to separate and code activities and participation,^39^ and the coding of P&O devices lacks specificity.^17^ Still, by implementing the ICF on a conceptual level (if not the coding) in clinical practice, P&O professionals would not only speak the language universal for rehabilitation but also contribute to its future development.

## Conclusion

A model, the POP model, is proposed that relates the concepts of ICF to the clinical P&O process. The POP model could support the implementation of ICF in the P&O field, thereby facilitate communication with other rehabilitation professionals and underline a holistic and patient-centred approach in clinical practice.
